# Rifaximin reduces gut-derived inflammation in severe acute pancreatitis: an experimental animal model and randomized controlled trial

**DOI:** 10.1128/spectrum.01299-25

**Published:** 2025-09-08

**Authors:** Yao-yu Zou, Bing-jun Yu, Cong He, Ling Ding, Xin Xu, Jian-hua Wan, Yu-peng Lei, Xin Huang, Hui-fang Xiong, Wen-hua He, Ling-yu Luo, Liang Xia, Nong-hua Lv, Yin Zhu

**Affiliations:** 1 Department of Gastroenterology, Jiangxi Provincial Key Laboratory of Digestive Diseases, Jiangxi Clinical Research Center for Gastroenterology, Digestive Disease Hospital, The First Affiliated Hospital, Jiangxi Medical College, Nanchang University 47861, Nanchang, Jiangxi, China; Ocean University of China, Qingdao, China

**Keywords:** acute pancreatitis, rifaximin, gut microbiota, systemic inflammatory response syndrome

## Abstract

Severe acute pancreatitis (SAP) is characterized by systemic inflammation and intestinal barrier dysfunction and is often associated with gut microbiota dysbiosis. Rifaximin, a gut-specific non-absorbable antibiotic, is known to modulate the gut microbiota. Here, we investigated rifaximin’s effects and mechanisms in SAP using murine models and a single-center, open-label, randomized controlled trial (Chinese Clinical Trial Registry: ChiCTR2100049794). In mice, rifaximin attenuated pancreatic injury and systemic inflammation and altered gut microbiota composition by decreasing mucin-degrading genera such as *Akkermansia* (*P <* 0.05). These protective effects persisted in antibiotic-treated and germ-free mice, suggesting mechanisms not solely dependent on gut microbiota modulation. In patients with predicted SAP (*n*  =  60), rifaximin significantly reduced systemic inflammation compared with controls. WBC decreased from a median of 11.50 × 10⁹/L (IQR 8.76–15.68) to 8.49 × 10⁹/L (6.93–10.20; *P* = 0.04) and TNF-α from 15.05 pg/mL (12.73–19.75) to 11.00 pg/mL (8.74–15.40; *P* = 0.009). However, the incidence of culture-confirmed infection did not differ between the rifaximin and control groups (13.3% vs. 13.3%; RR, 1.00; 95% CI, 0.28–3.63). Adverse events were comparable between groups. Metagenomic analyses revealed suppression of mucin-degrading bacteria (e.g., *Akkermansia*, *Bacteroides fragilis*, and *Hungatella hathewayi*) (*P <* 0.05) and reductions in mucin-degrading carbohydrate-active enzymes, including sialidases and fucosidases. In conclusion, among patients with predicted SAP, rifaximin did not reduce culture-confirmed infectious complications within 90 days after randomization compared with standard care, despite significant improvements in systemic inflammatory markers and selected fecal microbiome features. Larger randomized controlled trials are warranted to validate these findings.

**IMPORTANCE:**

Although rifaximin has been used to target gut-derived inflammation in other contexts, its role in SAP remains largely unexplored. In this study, rifaximin treatment was associated with reduced pancreatic injury and systemic inflammation in both murine models and patients with predicted SAP. Treatment also led to changes in gut microbial composition, notably a decrease in mucin-degrading taxa. Importantly, similar protective effects were also observed in antibiotic-treated and germ-free mice, indicating that rifaximin may act via microbiota-dependent and host-directed pathways. These findings offer novel insights into the gut-pancreas axis and suggest that rifaximin holds therapeutic potential by modulating gut microbial composition and host inflammatory responses in SAP.

## INTRODUCTION

Acute pancreatitis (AP) is a common abdominal emergency characterized by damage to pancreatic acinar cells secondary to premature activation of pancreatic enzymes caused by different etiologies, with or without organ dysfunction (1). Most attacks of AP are self-resolving, while patients with severe acute pancreatitis (SAP) have an aggressive onset and rapid progression, with a high mortality rate of up to 20% (2). Gut dysfunction is common in AP, especially SAP (3), which has been proven to be associated with adverse outcomes in AP (4, 5). Although the mechanisms leading to gut dysfunction in AP are complicated, early gut barrier dysfunction is thought to be the main cause (6). Gastrointestinal microecology, namely the biologic barrier, could be transformed into a pathogenic state, which is called microbiota dysbiosis, when interfered by the inflammatory stress during AP (7). Gut microbiota dysbiosis can disrupt the integrity of the intestinal mucosal barrier, facilitating the translocation of gut bacteria to the bloodstream and the pancreas through the compromised intestinal mucosa, leading to infections (8, 9). Gut-derived infections can worsen primary systemic inflammatory response syndrome (SIRS) and contribute to multiple organ dysfunction syndrome (MODS), often referred to as the “second hit” in AP (10). Therefore, regulating the gut microbiota may be a promising therapeutic approach for modulating systemic inflammation and improving clinical outcomes in AP.

Several treatments targeting gut microbiota dysbiosis have been studied, including enteral probiotics (11), prophylactic antibiotics (12), fecal microbiota transplantation (FMT) (13), and selective decontamination of the digestive tract (SDD) (14), among others (7). Oral administration of indigestible antibiotics can suppress the potential pathogens in the upper digestive tract, which enables SDD to effectively alleviate the extent of intestinal microbiota dysbiosis and reduce the bacterial translocation rate in AP patients (7). However, whether SDD should be supported in AP patients remains controversial in guidelines (15, 16), since the notion of antibiotic resistance induced by SDD must be considered (17). Rifaximin is an oral non-systemic gastrointestinal-targeted antibiotic that exerts its bactericidal effect by inhibiting bacterial RNA synthesis. It is characterized by minimal absorption into the bloodstream and no increase in secondary infections (18, 19). In addition to its antibiotic property, rifaximin has been well documented to effectively alter the gut microbiota and reduce circulating gut-derived endotoxin (20, 21).

Here, we evaluated the protective effects of rifaximin in a SAP mouse model and explored its potential mechanisms. Additionally, a single-center randomized controlled trial was conducted to preliminarily assess whether prophylactic rifaximin administration could reduce the incidence of infectious complications in AP, potentially enhancing patient outcomes and assessing safety. In particular, shotgun metagenomic sequencing of fecal samples was conducted in patients with AP to evaluate the effect of rifaximin on gut microbiota.

## MATERIALS AND METHODS

### Animal experiments with AP

#### Animals and experimental model

Balb/c wild-type mice (age 6 weeks) were purchased from Hunan SJA Laboratory Animal Co. Ltd. (Hunan, China) and housed under standard conditions (temperature: 22 ± 2℃; relative humidity, 50±5%; and a 12/12 h light/dark cycle). Germ-free (GF) mice were purchased from Shanghai SLAC Laboratory Animal Co., Ltd. (Shanghai, China). While GF mice were kept in sterile plastic isolators, other animals were housed under controlled environmental conditions. The mouse feeding regimen and experimental procedures were approved by the Institutional Animal Care and Use Committee of the First Affiliated Hospital of Nanchang University and complied with the national and international guidelines for the care and use of laboratory animals (Animal Ethics License Number: CDYFY-IACUC-202301QR015).

The induction of SAP was conducted following a previously described protocol by administering daily intraperitoneal injections of bombesin (100 µg/kg; Genscript Biotech Corporation, China) for seven consecutive days, followed on the eighth day by hourly intraperitoneal injections of caerulein (50 µg/kg; AnaSpec, Fremont, CA, USA) for 10 doses (22). The control group was intraperitoneally injected with the same dose of saline.

To explore the effects of rifaximin in AP, mice in the Cer + Bom + Rif and Control + Rif groups were administered rifaximin (250 mg/kg) intragastrically twice a day for eight days (ALFASIGMA S.p.A). All animals were sacrificed 12 hours after the last injection. Peripheral blood, pancreatic, and cecal contents were collected.

#### Construction of pseudogerm-free mice

The antibiotic (Abx) groups were administered broad-spectrum antibiotics (ampicillin 1 g/L [Sigma]; neomycin sulfate 1 g/ L [Sigma]; metronidazole 1 g/L [Sigma]; and vancomycin 0.5 g/L [Sigma]) in drinking water for 4 weeks as previously described.

#### Histopathological and immunohistochemical analysis (IHC)

Pancreatic tissue was fixed, dehydrated, and embedded in paraffin, and 5 µm sections were stained with hematoxylin and eosin (H&E). Pancreatic damage was assessed by two pathologists blinded to the treatment groups. Briefly, the evaluation of pancreatic pathology included four categories: edema, inflammatory cell infiltration, and necrosis (23). The infiltration of neutrophils was evaluated by IHC staining using anti-myeloperoxidase (MPO) rabbit polyclonal antibody (1:200, Abcam, ab9535).

#### Measurements of serum amylase, lipase, and interleukin (IL)-6

The activities of serum amylase and lipase were measured using commercial kits (IDEXX Laboratories, Inc., America) according to the manufacturer’s protocols. The levels of IL-6 were detected by a commercial kit (Serum multi-factor detection kit, Merck Millipore, Germany).

#### Quantification RT-PCR

Total RNA was extracted using the TRIzol Reagent (Servicebio, Wuhan, China). RNA concentration was determined using a nanophotometer (Stuttgart, Germany) and reverse transcribed using a complementary DNA conversion kit (Genesand, Beijing, China). qRT-PCR was performed using SYBR Green Master Mix (Genesand, Beijing, China) on the Line-Gene 9600 Plus Real-Time PCR System (Shanghai, China). The specific primers of relative genes are listed in Table S1.

#### 16S rRNA gene sequencing

The cecal contents obtained from mice were stored at −80°C until use. Microbial DNA from cecal contents was isolated, and the 16S rRNA was amplified, targeting the hypervariable region. The purified amplicon sequences were detected using Illumina MiSeq (PE300). The data were analyzed by Majorbio Bio-Pharm Technology Co. Ltd. (Shanghai, China).

### Clinical study of rifaximin in individuals with AP

#### Study design and participants

This prospective, single-center, open-labeled, randomized controlled trial was conducted at the Department of Gastroenterology, First Affiliated Hospital of Nanchang University, between August 2022 and November 2022 to assess the efficacy and safety of rifaximin in addition to standard care on AP patients. Each eligible patient was randomly assigned from a sequence of 60 participants generated using SPSS to either the rifaximin or control group in a 1:1 ratio. Medical records were traced and reviewed, and eligibility was determined by inclusion and exclusion criteria. The study was approved by the Ethics Committee of the First Affiliated Hospital of Nanchang University (2020030) and registered on the China Clinical Trials Center website (http://www.chictr.org.cn, registration number: ChiCTR2100049794) before enrollment commenced.

Patients diagnosed with AP (24) aged 18 to 70 years and SAP or predicted SAP (Acute Physiology and Chronic Health Evaluation II [APACHE II] score ≥8) without the use of antibiotics within 14 days were eligible for inclusion. The time from AP onset to enrollment was restricted to 72 h. Patients were excluded if they had post-endoscopic retrograde cholangiopancreatography pancreatitis, were pregnant or lactating women, had severe multi-organ failure (MOF) judged by physicians to be near death, a history of chronic pancreatitis, or allergy to rifaximin or other rifamycin analogs. Patients with a known history of malignancy, severe cardiovascular, respiratory, renal, or hepatic diseases, gastrointestinal bleeding, intestinal obstruction, or severe intestinal lesions were also excluded.

#### Interventions

All participants underwent interventions after enrollment and signing the informed consent. Patients in the rifaximin group were administered 200 mg rifaximin (ALFASIGMA S.p.A) thrice daily based on conventional therapy for 14 days starting on the second day of enrollment, and patients in the control group were administered only conventional therapy. For patients who died within 14 days, the drug was taken until death. For patients discharged within 14 days, it was continued after discharge. The use of prebiotics, probiotics, lactulose, and mannitol was prohibited, and prophylactic antibiotics were not recommended.

#### Randomization

A randomization sequence for 60 participants, with an equal allocation ratio of 1:1, was generated using SPSS by an independent statistician who was not involved in the clinical trial execution. The allocation concealment method used was the sequentially numbered, sealed, opaque envelope technique. Each participant received a study number upon enrollment, and an allocator (not involved in the study) assigned participants to either the rifaximin group or the control group based on the randomization sequence.

#### Clinical outcomes

The primary endpoint was the incidence of infectious complications diagnosed by culture during 90 days after randomization. Infectious complications included infected pancreatic necrosis (IPN), septicemia, and ascitic fluid infection. Multiple infections were considered to be one endpoint. Secondary clinical outcomes included infectious complications diagnosed by culture or clinical signs, other site infections (pneumonia, urinary tract infection, and biliary tract infection diagnosed by culture), use of intravenous antibiotics, complications and prognostic indicators (mortality, incidence of new-onset organ failure [OF], new-onset abdominal compartment syndrome [ACS], deterioration of intra-abdominal hypertension [IAH], local complications, severity of AP, length of hospital stay, length of hospital stay, and hospitalization charges), inflammatory indicators (white blood cell count [WBC], C-reactive protein [CRP], procalcitonin [PCT], interleukins [IL]-6, neutrophil percentage [NE], and tumor necrosis factor [TNF]-α). Definitions of the endpoints are detailed in Table S2.

Patients were followed during their hospital stay, and a follow-up was done at 90 days post-enrollment via clinical or telephone visits to assess readmission, mortality, and adverse events. Epidata software was used by a researcher for data collection. After dual verification of key variables by two researchers, the unedited data were exported for statistical analyses.

#### Sample collection and metagenomic sequencing

Fresh fecal samples were collected on day 1 (before treatment) and day 14 (after treatment). A total of 104 paired samples (52 per group) were obtained—fewer than planned due to patient withdrawal, early discharge, or lack of bowel movements. Samples were immediately frozen at −80°C for metagenomic sequencing. Total bacterial DNA was extracted, and sequencing was performed using the MGISEQ-T7 platform (OE Biotech Co., Ltd.). High-quality data were obtained by removing splice contamination, low-quality sequences, and host gene contamination from the raw data.

### Statistical analysis

Mice were randomly assigned to each group. The data were expressed as means and standard deviations. Differences between two groups with normal distributions were assessed by Student’s *t*-test, and one-way analysis of variance (ANOVA) followed by Bonferroni’s test was used to compare differences between more than two groups. Two-way ANOVA was used to compare the multi-factor differences between the two groups.

Clinical continuous data were presented as means and standard deviations or as medians and interquartile ranges, depending on their normality, and were analyzed using the Student’s *t*-test or Mann–Whitney *U* test, as appropriate. Categorical data were reported as numbers and percentages and were analyzed using the chi-square (χ²) test or Fisher’s exact test. Comparisons of endpoints were based on the intention-to-treat (ITT) population and expressed as relative risk (RR) with corresponding 95% confidence intervals (95% CI). All tests were two-tailed, and significance was set at *P* < 0.05. The statistical tests were carried out using GraphPad Prism software version 8.0.

For microbiome analyses, the observed Chao1 index was calculated to evaluate α-diversity of gastric microbiota. The Wilcoxon rank-sum test was applied to assess differences between two groups, while the Kruskal-Wallis test followed by Dunn’s post hoc test was used for multiple-group comparisons. β-diversity of microbial communities was characterized and visualized through principal coordinate analysis (PCoA) based on Bray–Curtis distances, and statistical differences among groups were tested using permutational multivariate analysis of variance (PERMANOVA) with the adonis function.

For taxonomic and functional annotation, DIAMOND software (v0.9.7, e-value cutoff = 1e-5) was used to align gene sets against the NR and KEGG databases to obtain annotations, with taxonomic abundances calculated at phylum, genus, and species levels. Functional annotation of carbohydrate-active enzymes (CAZymes) was conducted using the hmmscan tool (v3.1) against the CAZy database. Differential abundance analyses for microbial operational taxonomic units (mOTUs) and CAZymes were conducted using the SIAMCAT tool, with *P*-values corrected by the Benjamini-Hochberg method to control the false discovery rate (FDR).

### STORMS checklist

This study has been completed according to the STORMS checklist (DOI: https://doi.org/10.5281/zenodo.15256085).

## RESULTS

### Rifaximin alleviated pancreatic injury and inflammatory response in SAP mice

**Fig 1:**
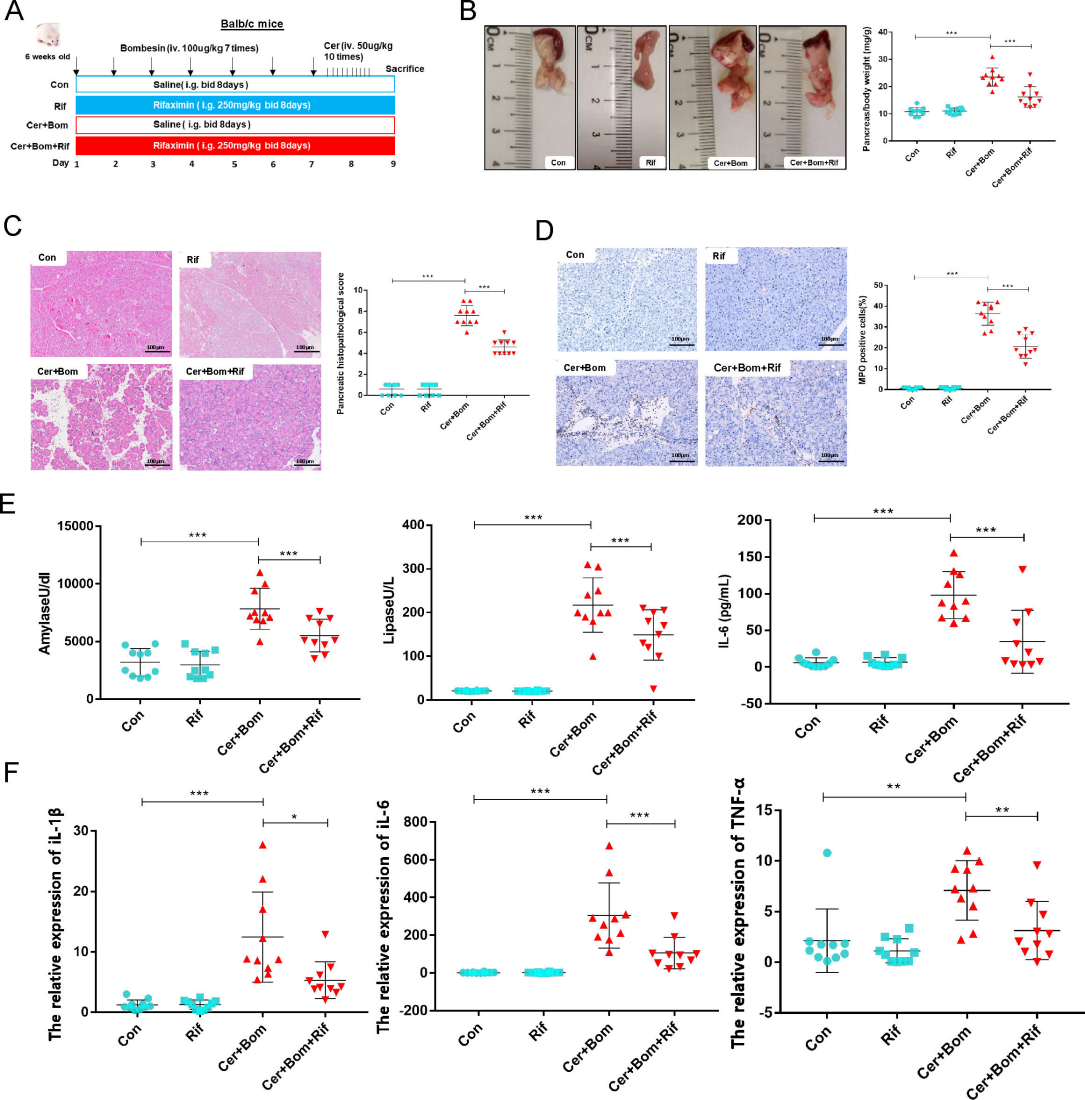
Rifaximin alleviated pancreatic injury and inflammatory response in SAP mice. (**A**) The schematic diagram of the modeling methods for the Rif group, Cer + Bom group, and Cer + Bom + Rif group. (**B**) Pancreatic weight ratios in the four groups. (**C**) H&E staining and pathological scores of pancreatic tissues in the four groups. (**D**) IHC staining and quantitation of MPO in the pancreatic tissue. (**E**) The serum levels of amylase, lipase, and IL-6. (**F**) The mRNA expression of proinflammatory cytokines in the pancreas, including IL-1β, IL-6, and TNF-α. Data are presented as mean ± SEM, *n* = 10 per group, *P < 0.05, **P < 0.01, and ***P < 0.001. SAP, severe acute pancreatitis; Rif, rifaximin; Cer, caerulein; Bom, bombesin; Con, control; MPO, myeloperoxidase.

First, we established a stable mouse model of SAP (Fig. S1). As shown in Fig. 1A, SAP is induced by co-administration of caerulein and bombesin, with prophylactic rifaximin administered to designated groups. SAP mice exhibited increased gross pancreas volume and pancreatic weight ratio, which were reduced by the prophylactic use of rifaximin (Fig. 1B). To investigate the effects on the pancreas in SAP mice, pancreas pathology was observed by H&E staining. Results showed that SAP mice displayed aggravated degrees of inflammatory cell infiltration, edema, and acinar necrosis compared to the control group. Nevertheless, rifaximin intervention significantly attenuated the histological injury in the pancreas of SAP mice, as evidenced by the histologic scores (Fig. 1C). The marked increase in neutrophil sequestration in the pancreas in SAP mice, as measured by myeloperoxidase (MPO) activity, was significantly downregulated in the rifaximin intervention group (Fig. 1D). Furthermore, rifaximin intervention downregulated serum amylase and lipase levels in SAP mice (Fig. 1E). The serum levels of inflammatory cytokines IL-6 were also decreased in the rifaximin intervention group compared to the SAP group, which indicates alleviation from systemic inflammation caused by SAP (Fig. 1E). And proinflammatory cytokines (IL-1β, IL-6, and TNF-α) were detected through qPCR of the pancreatic tissues. Results showed that high levels of proinflammatory cytokine factors were released in the SAP group. However, the prophylactic use of rifaximin alleviated pancreatic inflammation (Fig. 1F).

### Rifaximin alters abundance and structure of gut microbiota in SAP mice

**Fig 2:**
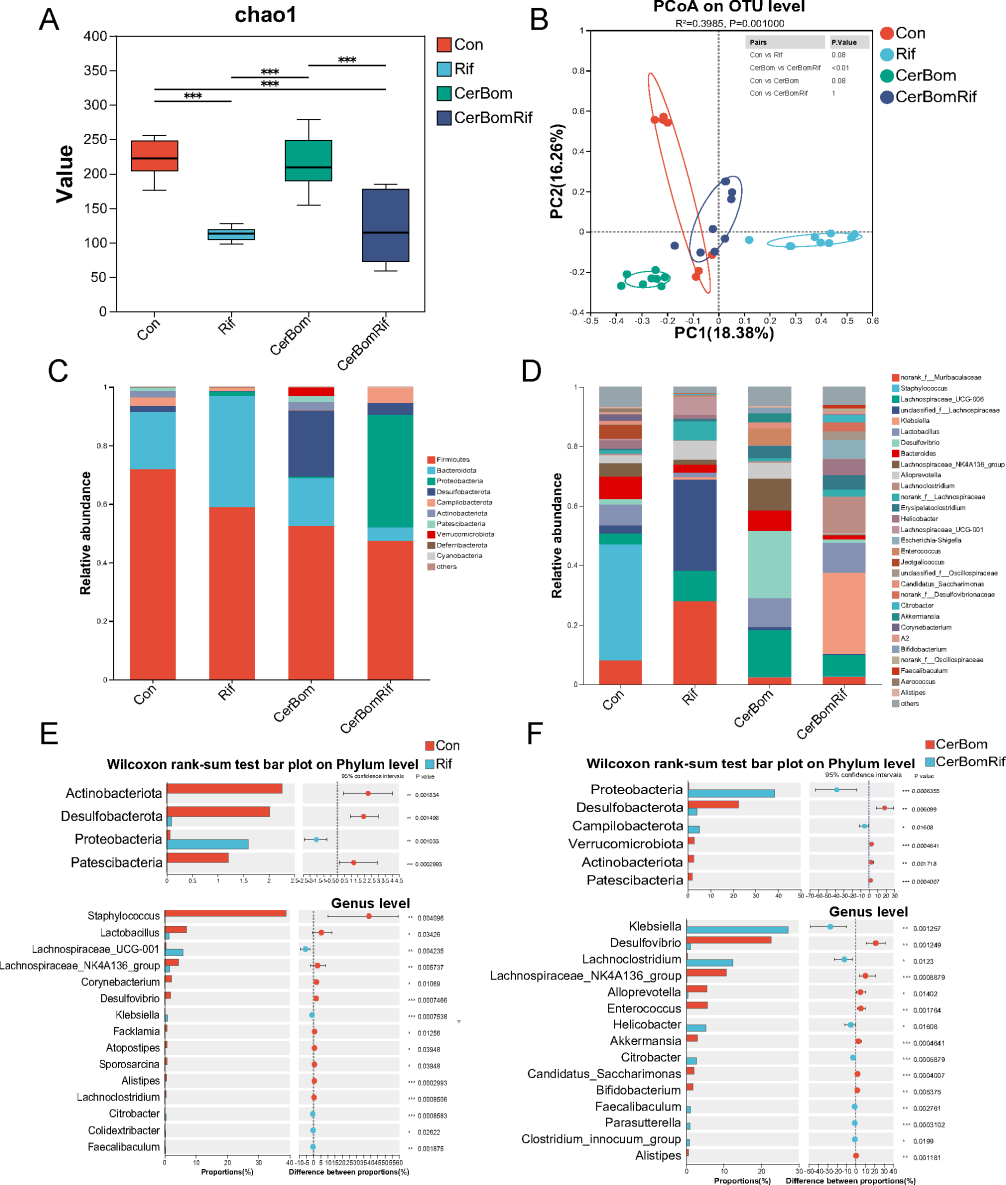
Bacterial abundance and community composition differences across Con group, Rif group, Cer + Bom group, and Cer + Bom + Rif group. (**A**) Alpha diversity illustrated by Chao1 index. (**B**) Beta diversity illustrated by PCoA based on Bray–Curtis distances. (**C**) Relative abundance of gut microbiota at the phylum level. (**D**) Relative abundance of gut microbiota at the genus level. (**E**) Wilcoxon rank-sum test identified the most differentially abundant phyla and genera between Con group and Rif group. (**F**) Wilcoxon rank-sum test identified the most differentially abundant phyla and genera between Cer + Bom group and Cer + Bom + Rif group. *P < 0.05, **P < 0.01, and ***P < 0.001. Rif, rifaximin; Cer, caerulein; Bom, bombesin; Con, control; PCoA, principal coordinate analysis.

After the experiments, fresh feces of mice were collected to analyze the gut microbiota using the 16S rRNA gene sequencing. We analyzed alpha diversity using the Chao1 richness index, which was significantly lower after rifaximin intervention in both normal and SAP mice (*P* < 0. 05) (Fig. 2A), confirming that the composition of the gut microbiota was affected by rifaximin. PCoA based on the Bray–Curtis distance showed a significant separation of the gut microbiota community structures of the rifaximin intervention group and the SAP group (Fig. 2B).

Then, we analyzed the differences in gut microbiota among the four groups at the phylum and genus levels (Fig. 2C through F). We mainly focused on where the gut microbiota was significantly affected after rifaximin intervention. At the phylum level, the abundance of *Proteobacteria* was significantly increased, and the abundance of *Actinobacteriota*, *Desulfobacteria*, and *Patescibacteria* was significantly decreased after rifaximin intervention in both normal and SAP mice (Fig. 2E and F). At the genus level, the abundance of *Klebsiella* and *Lachnoclostridium* was significantly increased, and the abundance of *Desulfovibrio*, *Alloprevotella*, *Enterococcus*, and *Akkermansia* was significantly decreased after rifaximin intervention in SAP mice (Fig. 2F).

### Rifaximin alleviated pancreatic injury and inflammatory responses in SAP mice independently of complete reliance on the gut microbiota

**Fig 3:**
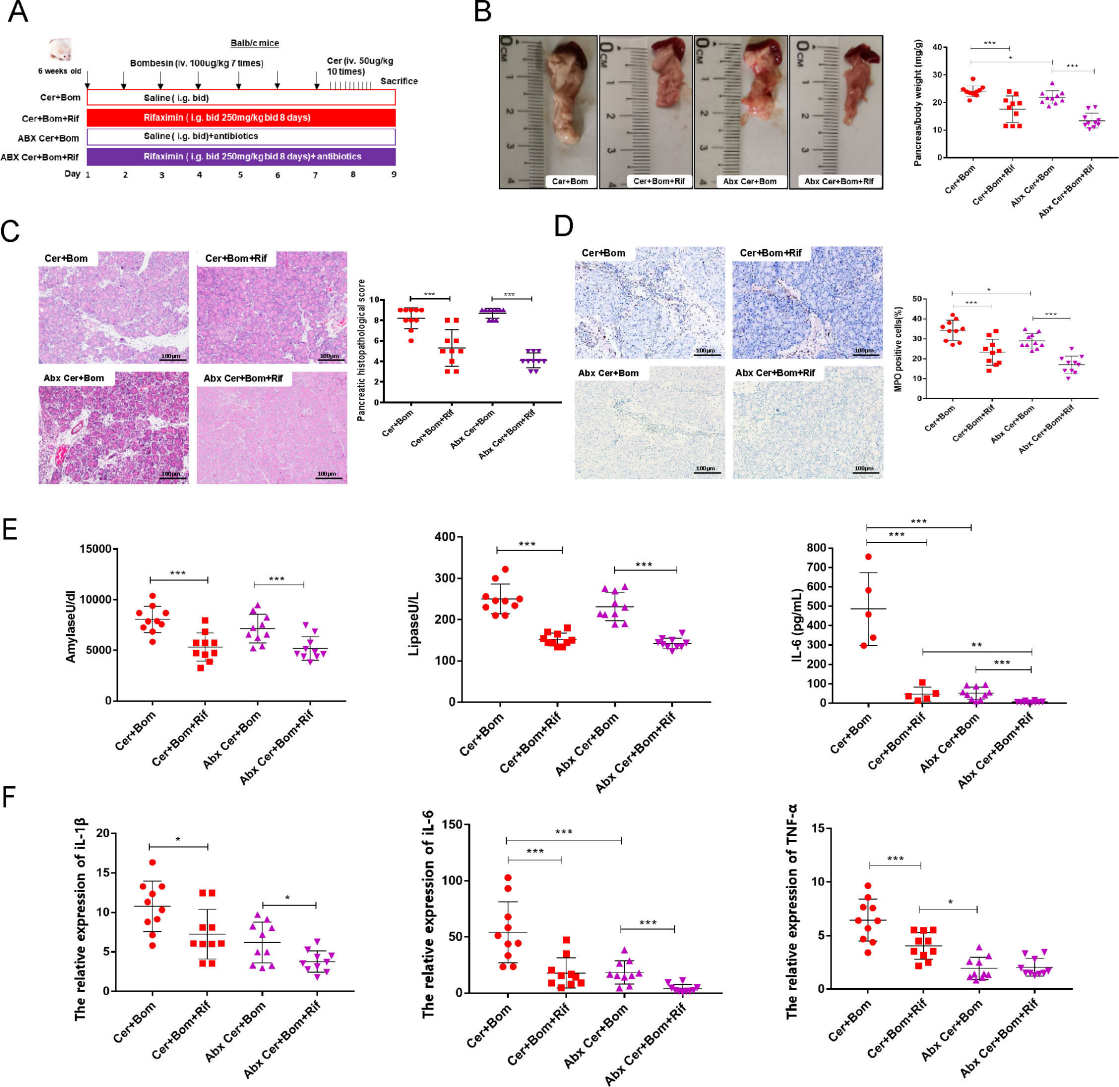
Rifaximin alleviated pancreatic injury and inflammatory response in SAP mice treated with Abx. (**A**) The schematic diagram of the modeling methods for the Cer + Bom group, Cer + Bom + Rif group, Abx Cer + Bom group, and Abx Cer + Bom + Rif group. (**B**) Pancreatic weight ratios in the four groups. (**C**) H&E staining and pathological scores of pancreatic tissues in the four groups. (**D**) IHC staining and quantitation of MPO in the pancreatic tissue. (**E**) The serum levels of amylase, lipase, and IL-6. (**F**) The mRNA expression of proinflammatory cytokines in the pancreas, including IL-1β, IL-6, and TNF-α. Data are presented as mean ± SEM, *n* = 10 per group, *P < 0.05, **P < 0.01, and ***P < 0.001. SAP, severe acute pancreatitis; Abx, antibiotics; Rif, rifaximin; Cer, caerulein; Bom, bombesin; Con, control; MPO: myeloperoxidase.

To investigate whether rifaximin attenuates SAP through the modulation of gut microbiota, we supplemented the drinking water of mice with broad-spectrum antibiotics (ampicillin, neomycin, metronidazole, and vancomycin) and established the animal models (Fig. 3A). After prophylactic administration of rifaximin, the pancreatic volume in SAP mice treated with Abx significantly reduced. The results of pancreatic weight ratio analysis were consistent with visual observations (Fig. 3B). Furthermore, in SAP mice treated with Abx, rifaximin intervention led to diminished pancreatic tissue pathological scores, pancreatic MPO activity, and lowered serum levels of amylase, lipase, and IL-6, as well as proinflammatory cytokines in the pancreas (IL-1β, IL-6, and TNF-α) (Fig. 3C through F). Consistent with the antibiotic-treated mice, GF mice with SAP exhibited reduced pancreatic injury and lower serum levels of amylase, lipase, and proinflammatory cytokines in the pancreas (IL-1β, IL-6, and TNF-α), following rifaximin intervention (Fig. S2). Together, our data suggested that the protective effect of rifaximin against SAP might be partly attributed to its effect on the gut microbiota.

### Rifaximin alleviates systemic inflammatory responses in patients predicted to SAP

**Fig 4:**
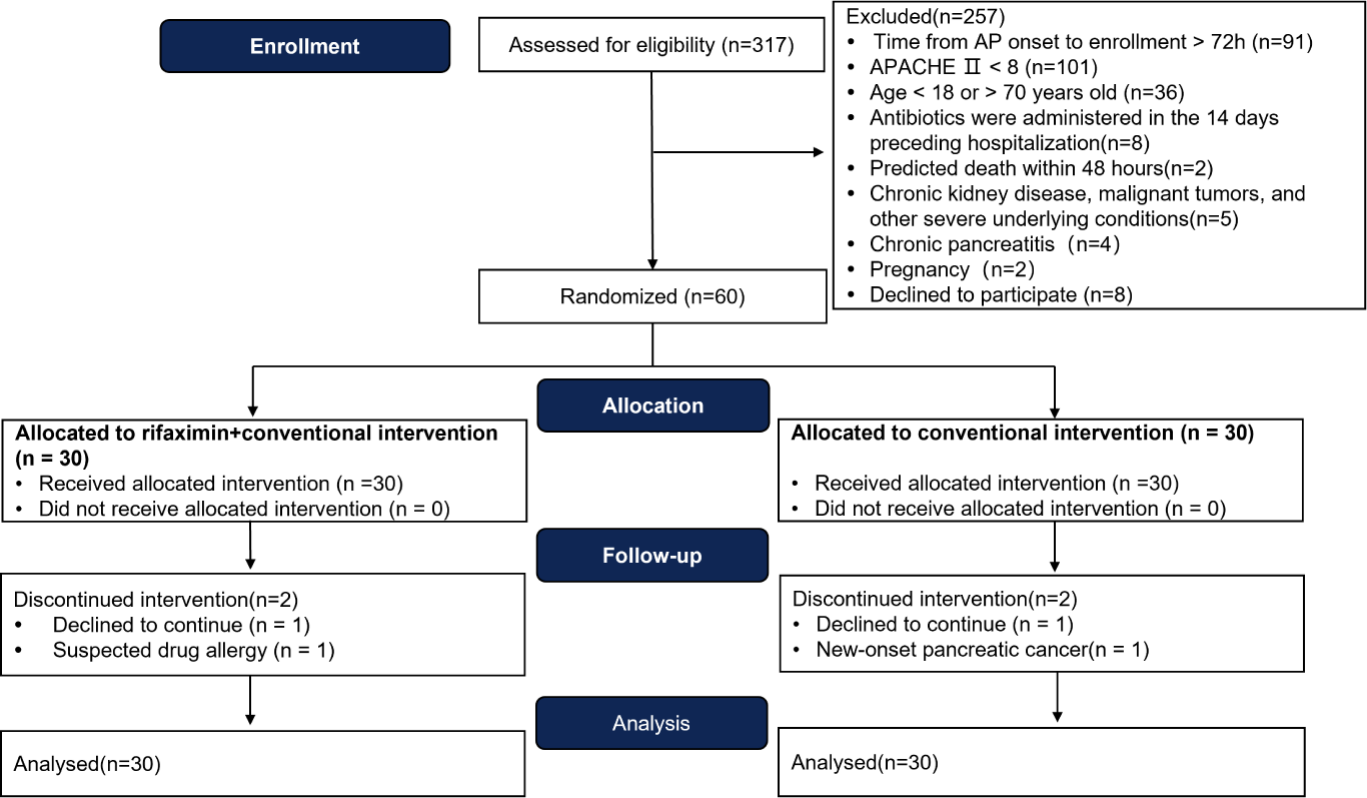
The flowchart of the clinical study. AP, acute pancreatitis.

To explore the clinical application of rifaximin, patients with AP (*n* = 60) were enrolled and randomly assigned to the rifaximin group (*n* = 30) or control group (*n* = 30) (Fig. 4). Four patients dropped out: two for poor compliance, one for suspected drug allergy, and one for new-onset pancreatic cancer. All analysis was performed based on the ITT principle. The baseline characteristics were not statistically different between the two groups (Table 1).

**TABLE 1:** Baseline characteristics of all patients^*a*^

	Rifaximin group (*n* = 30)	Control group (*n* = 30)	*P* value
Age, years	46.0 (32.8, 60.3)	48.0 (36.0, 53.0)	0.717
Sex			0.390
Male	20 (66.7%)	23 (76.7%)	
Female	10 (33.3%)	7 (23.3%)	
BMI, kg/m^2^	24.3 (23.3, 27.2)	26.1 (24.3, 27.5)	0.080
Etiology			0.603
Biliary	11 (36.7%)	9 (30.0%)	
Hyperlipidemia	10 (33.3%)	13 (43.3%)	
Alcohol excess	1 (3.3%)	2 (6.7%)	
Hyperlipidemia and Alcohol excess	6 (20.0%)	4 (13.3%)	
Biliary and Alcohol excess	0	1 (3.3%)	
IPMN	0	1 (3.3%)	
Idiopathic	2 (6.7%)	0 (0.0%)	
AP onset to hospital admission			0.530
≤24 h	14 (46.7%)	17 (56.7%)	
24–48 h	11 (36.7%)	7 (23.3%)	
48–72 h	5 (16.7%)	6 (20.0%)	
Smoker	13 (43.3%)	17 (56.7%)	0.302
Drinker	12 (40.0%)	9 (30.0%)	0.417
History of AP	8 (26.7%)	4 (13.3%)	0.197
History of hypertension	8 (26.7%)	8 (26.7%)	>0.999
History of diabetes	8 (26.7%)	5 (16.7%)	0.347
Admission clinical severity			
APACHE II score	10.5 (8.0, 12.2)	10.0 (8.0, 12.2)	0.822
SIRS score	2.0 (2.0, 3.0)	2.0 (2.0, 3.0)	0.882
MCTSI score	6.0 (4.0, 8.0)	4.0 (4.0, 6.0)	0.120
Organ failure^*b*^	21 (70.0%)	20 (66.7%)	0.781
Single organ failure			
Respiratory	13 (43.3%)	15 (50.0%)	0.605
Circulatory failure	0	0	
Renal	2 (6.7%)	1 (3.3%)	＞0.999
Multiple organ failure	6 (20.0%)	4 (13.3%)	0.488
Necrotizing pancreatitis	18 (60.0%)	19 (63.3%)	0.791
IAH/ACS	13 (43.3%)	14 (46.7%)	0.795

^*a*^ Data are presented as medians (interquartile ranges), or absolute numbers (proportions). IPMN, intraductal papillary mucinous neoplasm; AP, acute pancreatitis; BMI, body mass index; APACHE II score, Acute Physiology and Chronic Health Evaluation II score; SIRS score, systemic inflammatory response syndrome score; MCTSI, modified computed tomography severity index score; IAH, intra-abdominal hypertension; ACS, abdominal compartment syndrome.

^*b*^ Organ failure and severity were assessed according to the 2012 Atlanta Classification of AP.

We found no difference between the groups in the primary endpoint: both four cases occurred with infectious complications diagnosed by culture in the rifaximin group and in the control group (13.3%; RR, 1.00; 95% CI, 0. 28 to 3.63) (Table 2). The incidence of infectious complications, diagnosed by culture or clinical signs, was 20.0% in the rifaximin group and 33.3% in the control group, although there was no statistical difference (RR, 0.60; 95% CI, 0.25 to 1.44; *P* = 0.243), nor were there any significant differences between the groups regarding different infection sites (*P* > 0.05). The predominant site of infection in both groups was the bloodstream, and no ascitic fluid infection occurred.

**TABLE 2:** Primary and secondary endpoints^*a*^

	Rifaximin group (*n* = 30)	Control group (*n* = 30)	RR (95% CI)	*P* value
Primary endpoint:				
Infectious complications^*b*^ diagnosed by culture	4 (13.3%)	4 (13.3%)	1.00 (0.28–3.63)	＞0.999
Secondary endpoint:				
Infectious complications diagnosed by culture or clinical signs	6 (20.0%)	10 (33.3%)	0.60 (0.25–1.44)	0.243
IPN diagnosed by culture or clinical signs	2 (6.7%)	4 (13.3%)	0.50 (0.10–2.52)	0.667
IPN diagnosed by culture	2 (6.7%)	1 (3.3%)	2.00 (0.19–20.90)	＞0.999
IPN diagnosed by clinical signs	0	3 (10.0%)		0.236
Septicemia diagnosed by culture or clinical signs	6 (20.0%)	9 (30.0%)	0.67 (0.27–1.64)	0.371
Septicemia diagnosed by culture	3 (10.0%)	4 (13.3%)	0.75 (0.18–3.07)	＞0.999
Septicemia diagnosed by clinical signs	4 (13.3%)	5 (16.7%)	0.80 (0.24–2.69)	＞0.999
Pulmonary infection diagnosed by culture	3 (10.0%)	1 (3.3%)	3.00 (0.33–27.23)	0.605
Urinary tract infection diagnosed by culture	1 (3.3%)	0		＞0.999
Biliary tract infection diagnosed by culture	0	1 (3.3%)		＞0.999
Received intravenous antibiotics	18 (60%)	22 (73.3%)	0.82 (0.57–1.18)	0.273
Length of intravenous antibiotics	5.5 (0,10.0)	7.50 (0,18.0)		0.261
Local complications				0.908
None	4 (13.3%)	4 (13.3%)		
APFC	0	0		
PPC	8 (26.7%)	7 (23.3%)		
ANC	10 (33.3%)	8 (26.7%)		
WON	8 (26.7%)	11 (36.7%)		
Severity of AP^*c*^				0.850
MAP	3 (10.0%)	2 (6.7%)		
MSAP	7 (23.3%)	9 (30.0%)		
SAP	20 (66.7%)	19 (63.3%)		
New-onset organ failure	6 (20.0%)	9 (30.0%)	0.67 (0.27–1.64)	0.371
Multiple organ failure	4 (13.3%)	3 (10.0%)	1.33 (0.33–5.46)	＞0.999
Deterioration of IAH	2 (6.7%)	1 (3.3%)	2.00 (0.19–20.90)	＞0.999
New-onset ACS	4 (13.3%)	5 (16.7%)	0.80 (0.24–2.69)	＞0.999
Abdominal bleeding requiring intervention	1 (3.3%)	0		＞0.999
Portosplenomesenteric venous thrombosis	2 (6.7%)	1 (3.3%)	2.00 (0.19–20.90)	＞0.999
Pancreatogenic portal hypertension	1 (3.3%)	2 (6.7%)	0.50 (0.05–5.22)	＞0.999
Mechanical ventilation	6 (20.0%)	4 (13.3%)	1.50 (0.47–4.78)	0.488
Renal replacement therapy	3 (10.0%)	3 (10.0%)	1.00 (0.22–4.56)	＞0.999
Vasoactive drugs	6 (20.0%)	4 (13.3%)	1.50 (0.47–4.78)	0.488
PCD	2 (6.7%)	2 (6.7%)	1.00 (0.15–6.64)	＞0.999
Death	4 (13.3%)	2 (6.7%)	2.00 (0.40–10.11)	0.667
Length of ICU stay, days	24.5 (15.0, 28.0)	23.0 (10.75, 28.0)		0.894
Length of hospital stay, days	14.0 (8.0, 19.0)	16.5 (10.0, 28.3)		0.216
Total hospitalization charges, thousand yuan	36.5 (19.5, 67.3)	51.5 (22.3, 82.3)		0.408

^*a*^ RR, relative risk; CI, confidence interval; IPN, infectious pancreatic necrosis; APFC, acute peripancreatic fluid collection; PPC, pancreatic pseudocyst; ANC, acute necrotic collection; WON, walled-off necrosis; AP, acute pancreatitis; MAP, mild acute pancreatitis; MSAP, moderately severe acute pancreatitis; SAP, severe acute pancreatitis; IAH, intra-abdominal hypertension; ACS, abdominal compartment syndrome; PCD, Percutaneous drainage; ICU, intensive care unit.

^*b*^ Infectious complications included IPN, septicemia, and ascitic fluid infection.

^*c*^ Severity was assessed according to 2012 Atlanta Classification of AP. IAP that rebounded≥5 mmHg or increased≥20 mmHg in 1–7 days after randomization.

**Fig 5:**
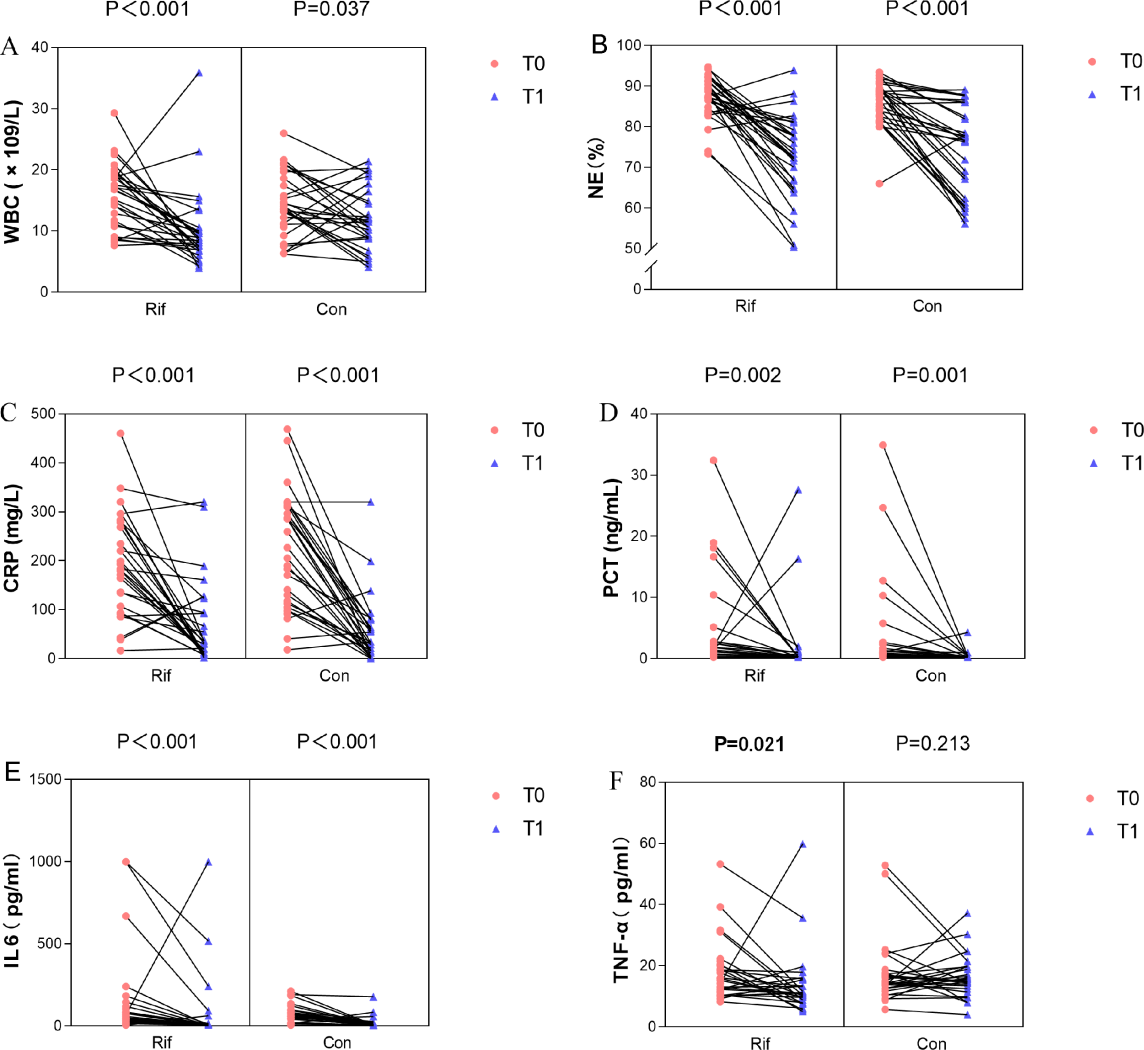
Comparison of the serum levels of inflammatory indicators before and after treatment in AP patients. (**A**) White blood cell (WBC), (**B**) neutrophilic granulocyte (NE), (**C**) C-reactive protein (CRP), (**D**) procalcitonin (PCT), (**E**) IL-6, and (**F**) TNF-α. AP, acute pancreatitis; Rif, rifaximin; Con, control; T0, time before intervention; T1, 15 days after intervention or the day of death or discharge (for patients who died or were discharged within 15 days).

Additionally, we compared inflammatory markers at baseline (T0) and 15 days after intervention (T1, or on the day of death or discharge if within 15 days). Reduction rates are shown in Fig. 5. Levels of WBC, NE%, CRP, PCT, and IL-6 significantly decreased at T1 compared with baseline in both groups (all *P*  <  0.05). However, TNF-α significantly decreased only in the rifaximin group (median reduction, 16.88%; IQR, −11.00 to 57.29; *P*  =  0.02), but not in the control group (median reduction, 4.54%; IQR, −36.49 to 23.13; *P*  =  0.21). Additionally, the rifaximin group showed a greater reduction in WBC (median reduction, 38.49%; IQR, 12.83 to 62.20; *P*  <  0.001) compared with controls (median reduction, 25.11%; IQR, −16.24% to 49.98%; *P*  =  0.04). At T1, WBC (median, 8.49  × 10^9^ /L; IQR, 6.93–10.20) and TNF-α (median, 11.00 pg/mL; IQR, 8.74–15.40) were significantly lower in the rifaximin group than in the control group (median, 11.50  × 10^9^ /L; IQR, 8.76–15.68 and median, 15.05 pg/mL; IQR, 12.73–19.75, respectively; both *P*  <  0.05; Table S3). Furthermore, other clinical outcomes, including OF, local complications, and hospital stay, were not significantly different between the two groups (Table 2).

There were two cases of *Candida* infections in the rifaximin group compared to one case in the control group. Additionally, there were two cases of multidrug-resistant bacteria infections in the rifaximin group and three cases in the control group (Table S4 and S5). One patient developed back pruritus after the third day of administering rifaximin, followed by a light red papule on the back, leading to discontinuation of the medication (Table S5). Considering the patient’s history of eczema, this was assessed clinically as an event that was probably related to the study medication. No serious adverse events were attributed to rifaximin. The 90-day follow-up of the patients is shown in Table S6.

### Rifaximin induces specific alterations in the abundance and function of the gut microbiota in patients predicted to SAP

**Fig 6:**
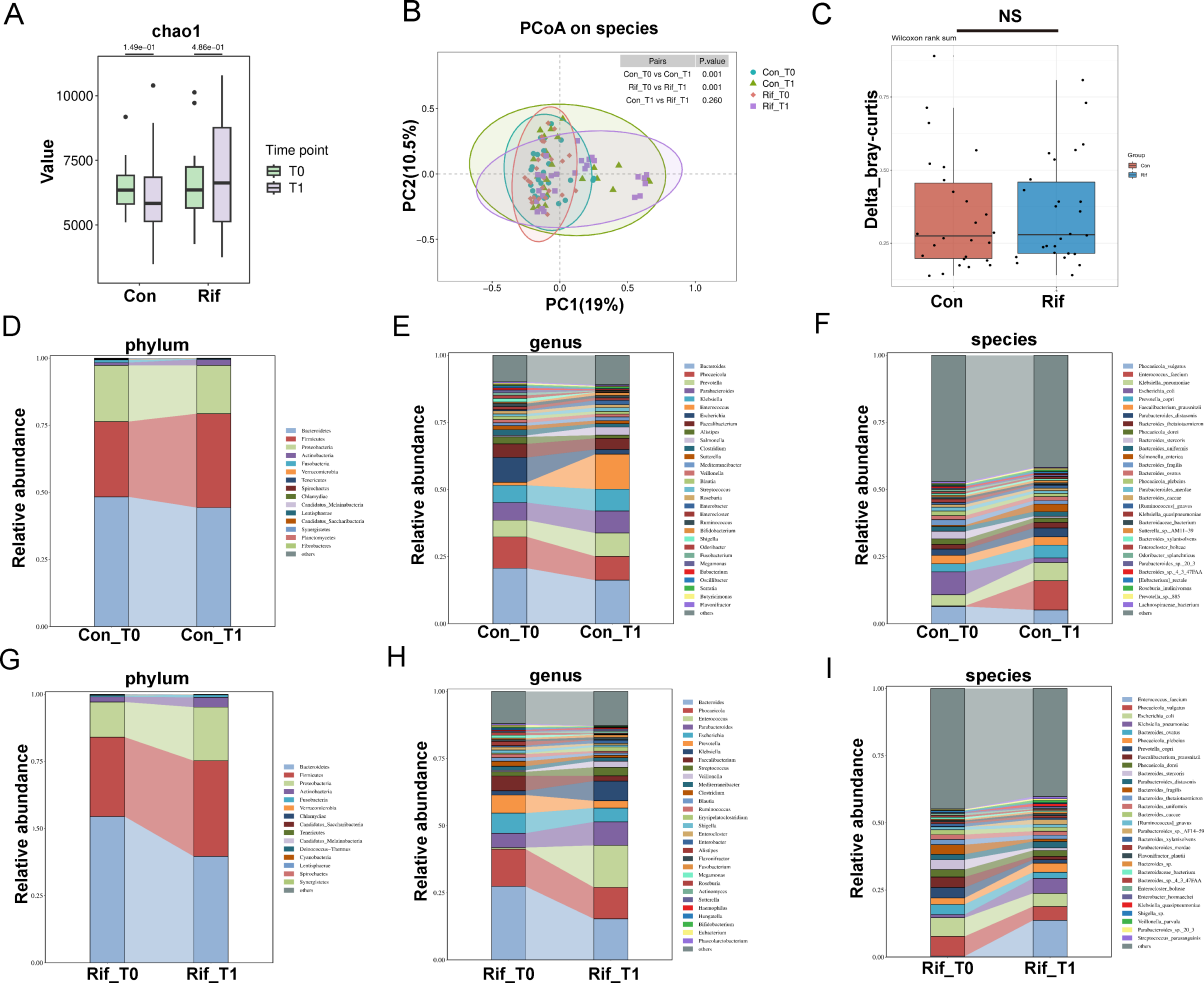
Bacterial abundance and community composition differences between the Con group and the Rif group in AP patients. (**A**) Alpha diversity illustrated by Chao1 index. (**B**) Beta diversity illustrated by PCoA based on Bray–Curtis distances. (**C**) The Bray–Curtis distances between Con group and Rif group were calculated before and after treatment. (**D**) Relative abundance of gut microbiota at the phylum level in Con group. (**E**) Relative abundance of gut microbiota at the genus level in Con group. (**F**) Relative abundance of gut microbiota at the species level in Con group. (**G**) Relative abundance of gut microbiota at the phylum level in Rif group. (**H**) Relative abundance of gut microbiota at the genus level in Rif group. (**I**) Relative abundance of gut microbiota at the species level in Rif group. AP, acute pancreatitis; Rif, rifaximin; Con, control; PCoA, principal coordinate analysis. T0, time before intervention; T1, 15 days after intervention or the day of death or discharge (for patients who died or were discharged within 15 days).

**Fig 7:**
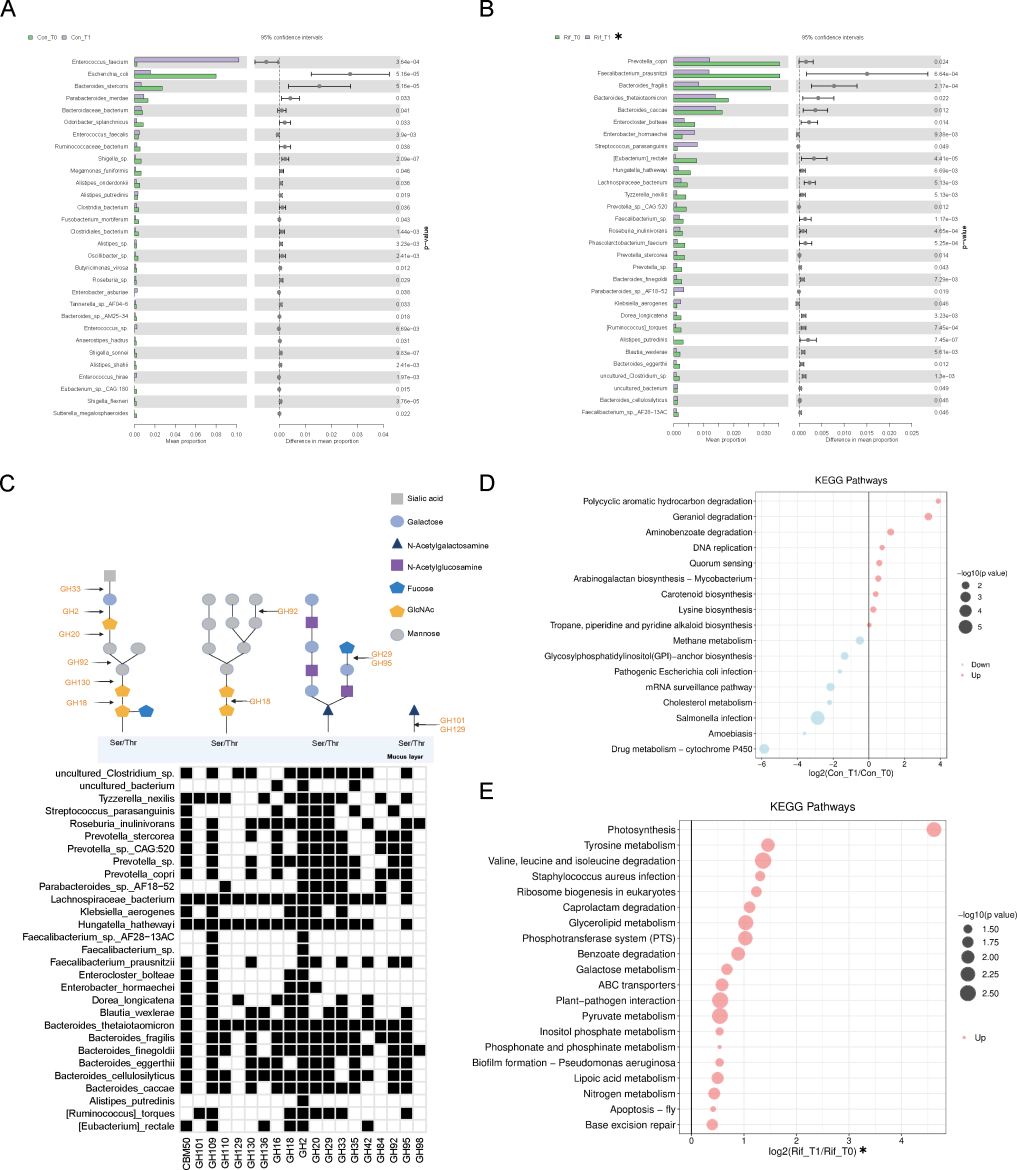
Rifaximin induces specific alterations in the abundance and function of the gut microbiota in patients predicted to SAP. (**A**) The top 30 differentially abundant bacterial species before and after treatment in the control group. (**B**) The top 30 differentially abundant bacterial species before and after treatment in the rifaximin group. (**C**) The mucin-degrading capacity of significantly contrasted species in feces based on CAZyme annotations of given MGS. The CAZyme families that degrade O-glycans of human mucins (black cells of heatmap) represent microbes with mucin-degrading CAZyme classification. (**D**) The predicted microbial functional annotation in the control group. (**E**) The predicted microbial functional annotation in rifaximin administration. *P* values in the plots showed *P* obtained from the Wilcoxon rank-sum test. *Indicates exclusion of bacteria or functional information with similar trends before and after treatment in both the rifaximin and control groups.

Stool samples were paired for two time points (T0 and T1). We also included 26 pairs of stool samples from both the rifaximin group and the control group to analyze the alterations in gut microbiota induced by rifaximin treatment, minimizing the influence of individual differences within groups. The alpha diversity as revealed by the Chao1 richness index did not change significantly among the two groups after intervention (Fig. 6A). PCoA based on the Bray–Curtis distance revealed different microbial communities before and after treatment both in the rifaximin and control groups, whereas there was no significant difference in microbiota changes between the two groups before and after treatment (Fig. 6B and 6C). Moreover, the composition of the microbiota was not significantly different between the two groups after treatment (Fig. 6B). The microbiota composition before and after treatment in the rifaximin and control groups was separately presented at different taxonomic levels (Fig. 6D through 6I). However, the abundance of gut microbiota had specific alterations at the phylum, genus, and species levels. At the phylum level, rifaximin decreased *Verrucomicrobia* and *Fibrobacteres* (*P* < 0.05) (Table S7). At the genus level, significant reductions in mucin-degrading genera, such as *Akkermansia* and *Hungatella*, were observed in the fecal samples (*P* < 0.05) (Table S8). At the species level, rifaximin suppressed the growth of *Bacteroides thetaiotaomicron*, *Bacteroides fragilis*, and *Hungatella hathewayi*. We assessed the mucin-degrading capacity of the top 30 species, which exhibited significant differences only after rifaximin intervention. This assessment was based on the carbohydrate-active enzyme (CAZyme) annotations of the species, including sialidases (GH33, GH101, and GH129) and fucosidases (GH29 and GH95). The CAZyme families that degrade O-glycans of human mucins are shown in Fig. 7C. Most gut species significantly decreased after rifaximin intervention are enriched with sialidases (GH33) and fucosidases (GH29, GH95), along with other mucin-degrading CAZymes (GH101, GH109, GH110, GH130, GH136, GH16, GH18, GH2, GH20, GH42, GH84, GH92, GH98, and CBM50). To assess changes in gut microbiota functions in AP, we calculated the relative abundance of KEGG pathways using metagenomic sequencing data. The rifaximin group showed significant differences in several KEGG categories compared to the control group. Notably, there were significant enhancements in energy metabolism pathways, including inositol phosphate, pyruvate, and galactose metabolism (Fig. 7D through E).

## DISCUSSION

This is the first comprehensive study evaluating rifaximin in AP using both preclinical and clinical models. In SAP mice, prophylactic rifaximin alleviated pancreatic injury and reduced local and systemic inflammatory responses. Similar anti-inflammatory effects were observed in patients with predicted SAP, where rifaximin significantly reduced systemic inflammatory markers, such as white blood cell count and TNF-α, demonstrating a good safety profile. However, no significant difference was observed in AP-related infectious complication rates. These findings suggest that rifaximin may modulate the early inflammatory response in SAP. In both animal and human fecal samples, rifaximin treatment was associated with reduced abundance of mucin-degrading taxa, such as *Akkermansia*. These microbial changes may reflect shifts in the gut environment related to inflammation or barrier function, although their mechanistic role requires further investigation.

In the early stages of AP, various proinflammatory mediators, including TNF-α, IL-1β, IL-6, and IL-8, are released into the bloodstream, amplifying the inflammatory response and leading to SIRS (25, 26). Severe and persistent SIRS inevitably results in multiple organ failures (27). Although the inflammatory cascade is considered to be the key mechanism of SAP, effective anti-inflammatory strategies are currently lacking. Classic anti-inflammatory drugs, such as glucocorticoids and non-steroidal anti-inflammatory drugs (NSAIDs), are not recommended for AP due to limited research and potential adverse events. Recently, numerous randomized controlled trials have shown that rifaximin, a poorly absorbed antibiotic, can reduce systemic IL-6 and TNF-α levels in cirrhotic patients (28–31). Since its introduction in Italy in 1987, rifaximin has been widely used to treat various clinical conditions, including common gastrointestinal diseases, such as cirrhosis-related complications (32), non-alcoholic fatty liver disease (33), inflammatory bowel disease (34), irritable bowel syndrome (35), and traveler’s diarrhea (36), exerting a local broad-spectrum antibiotic effect with minimal systemic side effects. This study is the first to comprehensively explore the effects of rifaximin on SAP mice and patients. Our results indicate that rifaximin can also reduce systemic IL-6 and TNF-α levels in AP, consistent with findings from other studies. A previous retrospective study on AP and rifaximin showed that patients receiving rifaximin had a significantly lower incidence of IPN and shorter median hospital stays compared to the control group (37); however, the study used historical controls and may be biased. However, our study did not find a significant effect of rifaximin on improving infectious complications in AP patients. Factors such as insufficient sample size, stricter definitions of infectious complications, the severity of AP at baseline, variations in the duration of administration, and routine treatments may influence the results.

This study is the first to use shotgun metagenomic sequencing and 16S rRNA high-throughput sequencing to explicitly identify changes in the gut microbiota in AP in response to rifaximin. Our previous studies have shown that changes in the structures of gut microbiota are associated with the development of AP (6, 38), and that regulating gut microbiota can alleviate AP (39, 40). Rifaximin, a poorly absorbed antibiotic, could regulate the composition of the gut microbiota (41, 42). Similarly, the results of 16S rRNA high-throughput sequencing in our study showed that rifaximin reduced α-diversity indices both in control and SAP mice compared to respective mice fed saline, indicating that rifaximin might inhibit the growth of some potentially pathogenic bacteria. The analysis of β-diversity showed significant differences in the bacterial community in SAP mice, which were strongly influenced by rifaximin treatment, as also reported in mouse models of irritable bowel disease (43). However, metagenomic sequencing of our clinical SAP cohort did not reveal significant changes in α-diversity, possibly due to routine clinical use of broad-spectrum intravenous antibiotics, inherent host and microbiome heterogeneity, and methodological differences between clinical and animal studies in terms of sampling sites, interventions, and disease management approaches (43–45).

Notably, rifaximin induced similar alterations in gut microbiota composition in both mouse and human fecal samples. Specifically, it reduced the abundance of mucin-degrading taxa, including *Verrucomicrobia* and *Akkermansia*, in SAP mice and patients predicted to develop SAP. Human metagenomic analysis further revealed decreased abundances of *Bacteroides fragilis*, *Bacteroides thetaiotaomicron*, and *Hungatella hathewayi*. These findings align with recent reports showing rifaximin-mediated suppression of opportunistic oral pathogens (*Akkermansia* and *Hungatella*) characterized by high sialidase activity, an enzyme involved in degrading gut mucin (29). Similarly, *Bacteroides fragilis* and *B. thetaiotaomicron* express mucin-degrading enzymes, including sialidases and fucosidases (46), that facilitate the breakdown of mucin glycans and disrupt intestinal barrier integrity, thus promoting local and systemic inflammation (47–49). . Therefore, these observations suggest that rifaximin might enhance the intestinal microenvironment, potentially supporting mucin production and gut barrier restoration, thereby reducing bacterial translocation and inflammation in AP. However, direct evaluation of the mucosal layer was beyond the scope of this study.

Notably, rifaximin exhibited protective effects in germ-free or broad-spectrum antibiotic-treated AP models, suggesting that its beneficial actions may extend beyond the modulation of gut microbiota alone. Studies have demonstrated that rifaximin acts as a potent agonist of intestinal pregnane X receptor (PXR), inhibiting NF-κB signaling and reducing nitric oxide (NO) production (50–52). Additionally, in colorectal and breast cancer models, rifaximin regulated cell proliferation and apoptosis through activation of PXR (53, 54). Moreover, rifaximin might exert anti-inflammatory effects by modulating TLR4 signaling pathways (55). Taken together, rifaximin’s protective role in AP may involve both microbiota-dependent and host-directed mechanisms, although the latter still requires further investigation through *in vitro* and *in vivo* studies.

This study has several strengths. First, we integrated preclinical animal experiments with a RCT in humans, providing consistent phenotypic and microbiota-related findings across species. To our knowledge, this is the first RCT assessing rifaximin as a therapy for AP. Second, by employing germ-free and pseudo-germ-free mouse models, we demonstrated that rifaximin’s therapeutic effects extend beyond modulation of gut microbiota alone, thereby adding robustness to our mechanistic insights. Several limitations should also be acknowledged. First, although randomization ensured balanced baseline therapies, residual confounding from intravenous antibiotic use cannot be entirely excluded. Second, our study did not fully characterize host-directed molecular mechanisms underlying the microbiota-independent actions of rifaximin in severe AP. Further research is required to clarify these pathways. Lastly, this study used an open-label design, which may introduce observation or reporting bias. Although we prespecified an objective primary endpoint (culture-confirmed infection) and performed analyses according to the intention-to-treat principle, residual bias cannot be fully excluded.

### Conclusion

In the single-center, open-label randomized trial involving patients with predicted SAP (APACHE II ≥ 8 at enrollment), rifaximin did not reduce culture-confirmed infectious complications during 90 days after randomization, despite improving inflammatory biomarkers and suppressing mucin-degrading taxa (e.g., *Akkermansia*, *Bacteroides fragilis*, and *Hungatella hathewayi*) associated with gut barrier impairment. Rifaximin also reduced pancreatic injury and systemic inflammation in murine SAP models. Larger, double-blind, adequately powered trials are warranted to confirm these findings.

## Data Availability

The data presented in the study are deposited in the Sequence Read Archive (https://www.ncbi.nlm.nih.gov/), accession number PRJNA1135945.

## SUPPLEMENTAL MATERIAL

Supplemental material 2 spectrum.01299-25-s0001.docx Tables S1 to S6; Fig. S1 and S2.

Supplemental material spectrum.01299-25-s0002.pdf Graphical abstract.

Table S7 spectrum.01299-25-s0003.xlsx Top 10 differentially abundant bacterial phyla after rifaximin treatment.

Table S8 spectrum.01299-25-s0004.xlsx Top 30 differentially abundant bacterial genera at the genus level after rifaximin treatment.

ASM does not own the copyrights to Supplemental Material that may be linked to, or accessed through, an article. The authors have granted ASM a non-exclusive, world-wide license to publish the Supplemental Material files. Please contact the corresponding author directly for reuse.
