# Supplementary material for: Rifaximin reduces gut-derived inflammation in severe acute pancreatitis: an experimental animal model and randomized controlled trial
Source: Microbiol Spectr. 2025 Sep 8;13(10):e01299-25. doi: 10.1128/spectrum.01299-25 (PMC12502533; doi:10.1128/spectrum.01299-25)
Supplement: Supplemental material 2 — Tables S1 to S6; Fig. S1 and S2. [file spectrum.01299-25-s0001.docx]

**Table S1.** primer sequence.

| **Target** | **Sense** | **Antisense** |
| --- | --- | --- |
| IL-1β | GCCACCTTTTGACAGTGATGAG | AAGGTCCACGGGAAAGACAC |
| IL-6 | CCACAACCACCTCAAGCACT | TAAGGCATCACAGTCCGAGTC |
| IL-10 | AGTTGGGGATTCGGTTGTTCT | AGTTGGGGATTCGGTTGTTCT |
| TNF-α | GACTAGCCAGGAGGGAGAAC | TGCTTTCTGTGCTCATGGTG |
| GAPDH | TGATGACATCAAGAAGGTGGTGAAG | TCCTTGGAGGCCATGTAGGCCAT |

**Table S2.** Comparison of the inflammatory indicators before and after intervention between the two groups.

| **Endpoint** | **Definition** |  |
| --- | --- | --- |
| IPN diagnosed by culture | A positive culture from pancreatic and/or peripancreatic necrosis obtained by drainage. |  |
| IPN diagnosed by clinical signs | In the first 14 days after onset of AP, the presence of gas configurations within pancreatic and peripancreatic necrosis on CT; after the first 14 days after onset, persistent organ failure in patients admitted to the intensive care unit or the persistence of two inflammatory variables (temperature >38.5°C or elevated CRP or WBC levels) during 3 consecutive days. | |
| Septicemia diagnosed by culture | A positive blood culture. | |
| Septicemia diagnosed by clinical signs | The presence of high fever (temperature >39°C), elevated PCT, and a high clinical suspected infection but a negative blood culture. | |
| Ascitic fluid infection | A positive culture of ascites. | |
| Pulmonary infection diagnosed by culture | A positive culture of sputum or BALF, excluding high clinical suspected colonization. | |
| Urinary tract infection diagnosed by culture | A positive urine culture, excluding high clinical suspected colonization. | |
| Biliary tract infection diagnosed by culture | A positive bile culture. | |
| New-onset organ failure | Organ failure occurring after randomization (not present at any time before randomization). | |
| Multiple-organ failure | Failure of two or more organs. | |
| Respiratory failure | PaO2/FiO2≤300, or requirement for mechanical ventilation. | |
| Circulatory failure | Circulatory systolic blood pressure<90 mmHg, despite adequate fluid resuscitation, or requirement for inotropic  catecholamine support | |
| Renal failure | Creatinine level>177 μmol/L after rehydration or new need for hemofiltration or hemodialysis. | |
| IAH | A sustained or repeated pathological elevation in IAP≥12 mmHg. | |
| ACS | A sustained IAP>20 mmHg (with or without an APP<60 mmHg) that is associated with new organ dysfunction/ failure. | |
| Deterioration of IAH | IAP that rebound≥5 mmHg or increases to≥20 mmHg within 7 days after randomization. | |
| New-onset ACS | ACS occurring after randomization (not present at any time before it), assessed for up to 4 weeks. | |

IPN, infectious pancreatic necrosis; AP, acute pancreatitis; CT; computed tomography; CRP, C-reactive protein; WBC, white blood cell; PCT, procalcitonin; BALF, bronchoalveolar lavage fluid; IAH, intra-abdominal hypertension; IAP; ACS, abdominal compartment syndrome; APP, intraperitoneal perfusion pressure.

**Table S3.** Comparison of the inflammatory indicators before and after intervention between the two groups.

|  | **Rifaximin group (n=30)** | **Control group (n=30)** | ***P* value** |
| --- | --- | --- | --- |
| Secondary endpoint: |  |  |  |
| WBC (×10^9^/L) |  |  |  |
| T0 | 16.70 (11.08, 19.06) | 13.66 (10.97, 18.90) | 0.329 |
| T1 | 8.49 (6.93, 10.20) | 11.50 (8.76, 15.68) | **0.042** |
| Δ (%) | 38.49 (12.83, 62.20) | 25.11 (-16.24, 49.98) | 0.095 |
| NE (%) |  |  |  |
| T0 | 88.05 (84.58, 91.15) | 87.95 (82.73, 89.70) | 0.429 |
| T1 | 72.90 (66.3, 79.7) | 76.70 (64.75, 84.25) | 0.575 |
| Δ (%) | 17.40 (9.15, 23.55) | 12.31 (3.87, 25.39) | 0.524 |
| CRP (mg/L) |  |  |  |
| T0 | 188.11 (103.55, 280.38) | 216.06 (110.84, 310.56) | 0.425 |
| T1 | 30.81 (9.57, 101.55) | 34.24 (13.81, 71.57) | 0.874 |
| Δ (%) | 86.81 (13.80, 93.21) | 82.01 (67.16, 93.58) | 0.705 |
| PCT (ng/mL) |  |  |  |
| T0 | 1.30 (0.54, 2.84) | 0.60 (0.25, 1.84) | 0.169 |
| T1 | 0.25 (0.25, 0.52) | 0.25 (0.25, 0.25) | 0.217 |
| Δ (%) | 66.06 (0, 89.95) | 54.53 (0, 88.24) | 0.919 |
| IL-6 (pg/ml) |  |  |  |
| T0 | 55.00 (31.13, 107.00) | 59.7 (37.00, 96.35) | 0.871 |
| T1 | 5.67 (2.82, 13.40) | 6.84 (4.56, 17.40) | 0.266 |
| Δ (%) | 87.54 (75.78, 94.92) | 83.99 (68.57, 92.22) | 0.321 |
| TNF-α (pg/ml) |  |  |  |
| T0 | 14.70 (11.00, 20.10) | 14.35 (12.58, 17.40) | 0.982 |
| T1 | 11.00 (8.74, 15.40) | 15.05 (12.73, 19.75) | **0.009** |
| Δ (%) | 16.88 (-11.00, 57.29) | 4.54 (-36.49, 23.13) | **0.049** |

WBC, white blood cell count; NE, percentage of neutrophils; CRP, C-reactive protein; PCT, procalcitonin; IL – 6, interleukin- 6; TNF-α, tumor necrosis factor-α. T0 denotes the day before intervention; T1 denotes 15 days after intervention or the day of death or discharge (for patients who died or discharged within 15 days); Δ (%) denotes the decline rates of each inflammatory index indicator before and after intervention.

**Table S4.** Comparison of the pathogenic microorganisms between the two groups.

|  | **Rifaximin group (n=30)** | **Control group (n=30)** | **RR (95% CI)** | ***P* value** |
| --- | --- | --- | --- | --- |
| Secondary endpoint: |  |  |  |  |
| Gram-negative bacteria |  |  |  |  |
| Klebsiella pneumoniae | 2 (50.0%) | 1 (25.0%) | 2.00 (0.28-14.20) | >0.999 |
| Gram-positive bacteria |  |  |  |  |
| Staphylococcus | 2 (50.0%) | 1 (25.0%) | 2.00 (0.28-14.20) | >0.999 |
| Enterococcus Faecium | 1 (25.0%) | 1 (25.0%) | 1.00 (0.09-11.03) | >0.999 |
| Fungi |  |  |  |  |
| Candida | 2 (50.0%) | 1 (25.0%) | 2.00 (0.28-14.20) | >0.999 |
| Multidrug-resistant bacteria | 2 (50.0%) | 3 (75.0%) | 0.67 (0.22-2.07) | >0.999 |

RR, relative risk; CI, confidence interval.

**Table S5.** Adverse Events, According to Study Group.

| **Event** | **Rifaximin group**  **(n=30)** | | **Control group**  **(n=30)** | **Severity (Mild/Moderate/Severe)** | **Relationship to Study Drug (Related/Possibly/Unrelated)** |
| --- | --- | --- | --- | --- | --- |
| Any event | | 3 (10%) | 3 (10.0%) | N/A | N/A |
| Rash | | 1 (3.3%) | 0 | Mild (1) | Possibly related (1) |
| Candida infections | | 2 (6.7%) | 1 (3.3%) | Moderate (1), Severe (1) (Rifaximin); Severe (1) (Control) | Unrelated (3) |
| Multidrug-resistant bacteria infections | | 2 (6.7%) | 3 (10.0%) | Moderate (1), Severe (1) (Rifaximin); Moderate (2), Severe (1) (Control) | Unrelated (5) |

**Note:** Severity and causal relationship were assessed by qualified senior clinicians. One patient experienced pruritus on the back on day 3 after initiating rifaximin therapy, subsequently developing mild erythematous papules, leading to study drug discontinuation. Given the patient's history of eczema, this adverse event was clinically assessed as possibly related to the study drug. All adverse events in the control group were considered unrelated to the study drug .

**Table S6.** The 90-day follow-up of the patients.

|  | **Rifaximin group**  **(n=26)** | **Control group**  **(n=28)** |
| --- | --- | --- |
| Fever | 0 | 2 (7.1%) |
| Bloating | 1 (3.8%) | 0 |
| Readmission | 2^a^ (7.7%) | 4^b^ (14.3%) |
| Recurrence of AP | 1 (3.8%) | 1 (3.6%) |
| Pancreatic endocrine insufficiency | 8 (30.8%) | 8 (28.6%) |

AP, acute pancreatitis. a: one patient needed readmission due to recurrence of AP and one due to proposed cholecystectomy; b: one patient needed readmission due to fever, one due to recurrence of AP, one due to the complications of walled-off necrosis, and one due to proposed cholecystectomy.


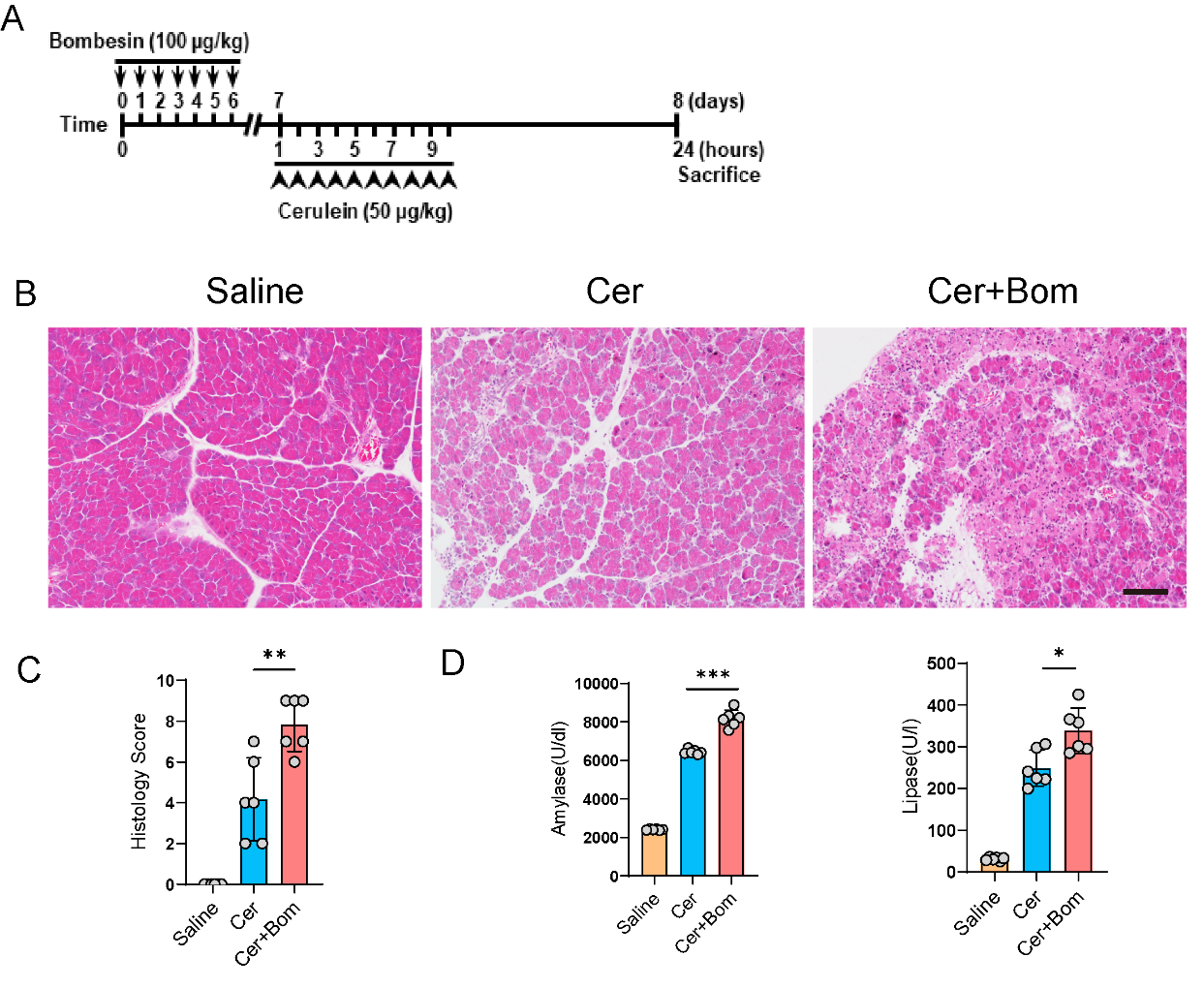


**Fig. S1.** Establishment of severe acute pancreatitis mouse model

(A) Experimental timeline of SAP model induction by Cer (50 µg/kg) and Bom (100 µg/kg).

(B) Representative H&E staining images of pancreatic tissues from Saline (control), Cer, and Cer + Bom groups. Scale bar, 25 µm.(C) Histological severity scores.(D) Serum amylase and lipase levels in each group. Data are presented as mean ± SEM. *P<0.05, **P<0.01, ***P<0.001. SAP: severe acute pancreatitis; Rif: rifaximin; Cer: Caerulein; Bom: bombesin.


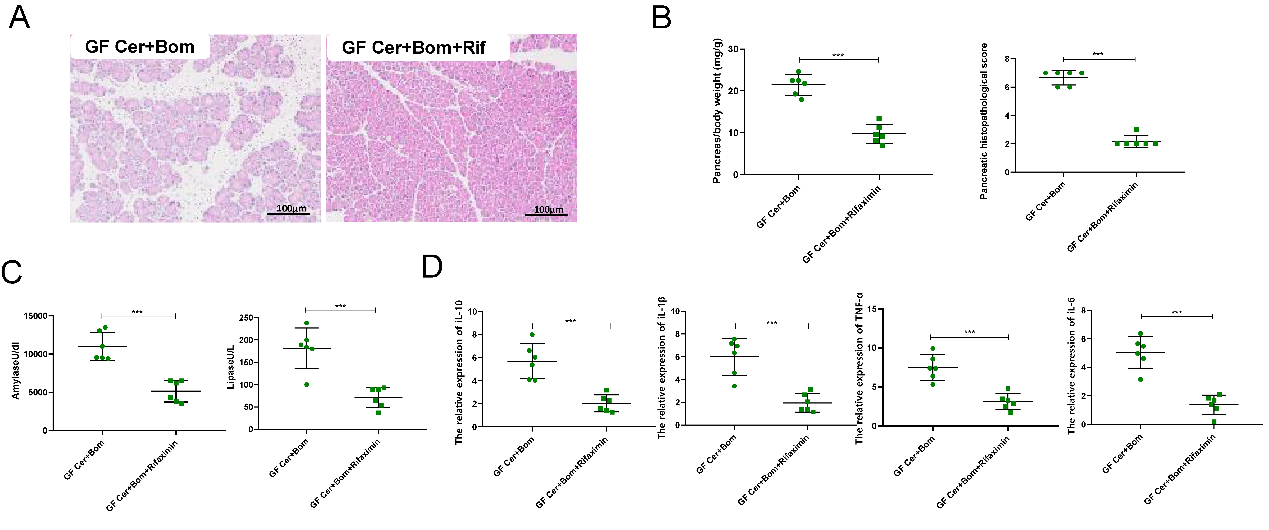


**Fig. S2.** Rifaximin alleviated pancreatic injury and inflammatory response in GF mice with SAP.

(A) H&E staining of pancreatic tissues in the GF Cer+ Bom group, and GF Cer+ Bom +Rif group. (B) Pancreatic weight ratios and pathological scores in the two groups. (C) The serum levels of amylase and lipase in the two groups. (D) The mRNA expression of proinflammatory cytokines in the pancreas of the two groups, including IL-10, IL-1β, TNF-α, and IL-6. Data are presented as mean ± SEM, n = 6 per group, ***P < 0.001. GF: germ-free; SAP: severe acute pancreatitis; Rif: rifaximin; Cer: Caerulein; Bom: bombesin.
