# Supplementary material for: Rifaximin reduces gut-derived inflammation in severe acute pancreatitis: an experimental animal model and randomized controlled trial
Source: Microbiol Spectr. 2025 Sep 8;13(10):e01299-25. doi: 10.1128/spectrum.01299-25 (PMC12502533; doi:10.1128/spectrum.01299-25)
Supplement: Supplemental material — Graphical abstract. [file spectrum.01299-25-s0002.pdf]

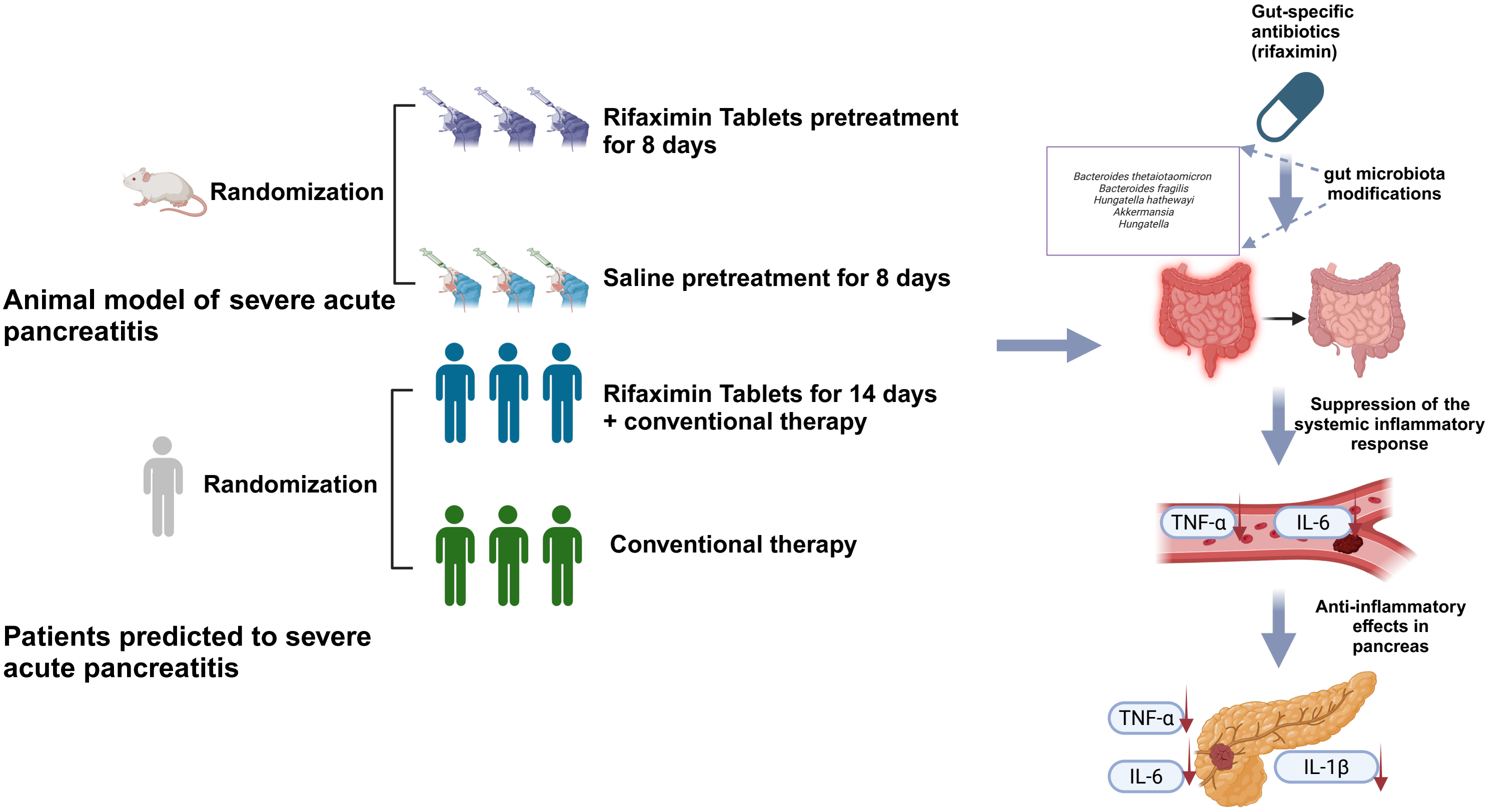

Gut-specific antibiotics (rifaximin)
Rifaximin Tablets pretreatment for 8 days
Randomization
Saline pretreatment for 8 days
Animal model of severe acute pancreatitis
Bacteroides thetaiotaomicron
Bacteroides fragilis
Hungatella hathewayi
Akkermansia
Hungatella
gut microbiota modifications
Rifaximin Tablets for 14 days + conventional therapy
Randomization
Conventional therapy
Patients predicted to severe acute pancreatitis
Suppression of the systemic inflammatory response
TNF-α
IL-6
Anti-inflammatory effects in pancreas
TNF-α
IL-6
IL-1β
